# Four-Factor Prothrombin Complex Concentrate vs Plasma in Patients on Vitamin K Antagonists With Gastrointestinal Bleeding or Needing a Gastrointestinal Procedure: A Retrospective Analysis of 2 Randomized Controlled Trials

**DOI:** 10.1016/j.acepjo.2025.100142

**Published:** 2025-04-16

**Authors:** Majed A. Refaai, Joshua N. Goldstein

**Affiliations:** 1 School of Medicine and Dentistry, University of Rochester, Rochester, New York, USA; 2 Department of Emergency Medicine, Massachusetts General Hospital, Boston, Massachusetts, USA

**Keywords:** 4-factor prothrombin complex concentrate, plasma, gastrointestinal bleeding, gastrointestinal surgery, vitamin K antagonist reversal

## Abstract

**Objectives:**

To examine the efficacy of 4-factor prothrombin complex concentrate (4F-PCC) compared with plasma in vitamin K antagonist (VKA)–treated patients with gastrointestinal (GI) bleeding or requiring a GI surgical/invasive procedure.

**Methods:**

A retrospective analysis was conducted on a subset of data from 2 prospective phase 3b randomized controlled trials of 4F-PCC or plasma for VKA reversal. Data from patients receiving VKA who experienced acute major GI bleeding or needed a GI surgical/invasive procedure within 24 hours were included in the analysis. Hemostatic efficacy, international normalized ratio (INR), and vitamin K–dependent coagulation factor (VKDF) restoration were analyzed.

**Results:**

In total, 171 patients were included in the analysis. Overall, hemostatic efficacy was rated excellent and good in 68 of 83 (81.9%) and 66 of 88 (75.0%) patients in the 4F-PCC and plasma treatment groups, respectively (odds ratio [OR], 1.52; 95% CI, 0.72-3.20). At 0.5 hours after infusion, 68.2% of patients treated with 4F-PCC achieved an INR of ≤1.3 compared with 0.0% of patients treated with plasma (68% difference; 95% CI, 57-79). Time to INR restoration from the start of infusion was significantly shorter for 4F-PCC than plasma (45 vs 1326 minutes, respectively; OR, 0.10; 95% CI, 0.07-0.14). All VKDF levels were significantly higher in the 4F-PCC group vs the plasma group within 3 hours from the start of infusion (all *P* < .002). Additional blood product use in the acute major bleeding study was comparable between both groups.

**Conclusion:**

4F-PCC was associated with a nearly immediate decrease in INR and rapid VKDF restoration compared with plasma in patients experiencing acute major GI bleeding or in need of GI surgery/invasive procedure. Yet, hemostatic efficacy was similar between the 2 groups, and therefore, larger studies might be needed to better understand patient outcomes.

**The Bottom Line**

Major gastrointestinal bleeding is a complication in patients on vitamin K antagonist therapy, for which both prothrombin complex concentrate and plasma are used. This retrospective analysis compared the efficacy of 4-factor prothrombin complex concentrate and plasma in patients with major gastrointestinal bleeding or undergoing gastrointestinal surgery. Four-factor prothrombin complex concentrate was associated with a near-immediate decrease in the international normalized ratio and faster restoration of vitamin K–dependent factors compared with plasma. Yet, hemostatic efficacy was similar between the 2 groups, and, therefore, larger studies might be needed to better understand patient outcomes.

## 1 Introduction

### 1.1 Background

Gastrointestinal (GI) bleeding is the most common type of major bleeding complication in patients on vitamin K antagonist (VKA) therapy.^1^, ^2^, ^3^ An analysis of phase 3 clinical trials comparing VKAs and direct oral anticoagulants showed that GI bleeding represented 33% of all major bleedings in VKA-treated patients.^4^ Patients with GI bleeding on VKA therapy are more likely to experience worse outcomes compared with GI bleeding patients without anticoagulation. In fact, in a recent multicenter real-world study of 2299 patients with GI bleeding, anticoagulation was associated with an 8-fold increased risk of all-cause 28-day mortality after adjusting for potential confounders.^5^ Therefore, rapid anticoagulation reversal and cessation of bleeding in this specific population may be critical for survival. This can be achieved by administering plasma or prothrombin complex concentrates (PCCs). Among PCCs, 3-factor PCC contains coagulation factors II, IX, and X, whereas 4-factor PCC (4F-PCC) also contains factor VII as well as proteins C and S.^6^^,^^7^ 4F-PCC can rapidly replenish vitamin K–dependent coagulation factors (VKDFs) in VKA-treated patients experiencing acute major GI bleeding or undergoing an urgent GI surgical or invasive procedure.^8^, ^9^, ^10^

### 1.2 Importance

As both PCC and plasma are administered for VKA reversal, one question is, which one is most commonly used? A recent retrospective analysis of real-world use of 4F-PCC and plasma for the management of oral anticoagulant-associated bleeding in US trauma hospitals revealed that plasma is used frequently for warfarin reversal (53% plasma vs 47% 4F-PCC).^11^ When asked, physicians who reported the choice of plasma commented on their experience and comfort with it, particularly for warfarin reversal.^11^ However, this study could not address which was superior. In order to compare these treatment options, the best approach may be to review data from clinical trials. Although none have been conducted exclusively for GI bleeding, 2 multicenter phase 3b randomized controlled trials (RCTs), which included GI bleeding among a range of indications, have shown that 4F-PCC is an effective alternative to plasma for urgent VKA reversal.^12^^,^^13^ These studies demonstrated that 4F-PCC achieved effective hemostasis in 72% to 90% of patients and rapidly restored international normalized ratio (INR) in patients presenting with major bleeding^12^ and in those needing an urgent surgical or invasive procedure.^13^ In a retrospective analysis of VKA-treated patients with acute/severe GI bleeding, 4F-PCC was also associated with smaller infusion volumes, shorter infusion times, and reduced time to the GI procedure than plasma.^14^ Another retrospective study found that 4F-PCC was associated with a shorter time to GI procedure and shorter emergency department (ED) and intensive care unit (ICU) length of stay (LOS).^15^ As a result of these and other studies, many current guidelines for GI bleeds in VKA-treated patients recommend 4F-PCC.^8^, ^9^, ^10^

### 1.3 Goal of This Investigation

The aim of the present study was to review patient-level data from RCTs to examine the efficacy of 4F-PCC compared with plasma in VKA-treated patients with GI bleeding or requiring a GI surgical/invasive procedure.

## 2 Methods

### 2.1 Study Design and Population

A retrospective analysis of data from 2 prospective phase 3b RCTs comparing 4F-PCC with plasma for VKA reversal was performed (NCT00708435 and NCT00803101).^12^^,^^13^ Full details of the study design of both RCTs have been published previously.^12^^,^^13^ Briefly, NCT00708435 was performed in 216 patients at 36 sites across the United States and Europe; NCT00803101 included 181 patients from 33 sites in the United States (18), Belarus (2), Bulgaria (4), Lebanon (2), Romania (1), and Russia (6). Patients were randomized (1:1) to receive either 4F-PCC (Kcentra, CSL Behring)^6^ or plasma IV infusion on day 1 of the study. Blood samples were drawn for INR and VKDFs at 0.5 hours, 1 hour, 3 hours, 6 hours, and 24 hours. The use of additional blood products was recorded up to 24 hours after the start of the product infusion; adverse events were recorded up to day 10, and serious adverse events up to day 45.^12^^,^^13^

This analysis included a subset of VKA-treated patients (≥18 years of age) with an elevated INR (≥2.0 within 3 hours before study treatment) experiencing an acute major GI bleeding event or in need of an urgent GI surgical or invasive procedure within 24 hours and receiving either 4F-PCC or plasma.

### 2.2 Endpoints

The primary endpoint of both studies was hemostatic efficacy (efficacy rating excellent or good). In the acute major bleeding RCT, for visible bleeding, excellent or good hemostasis was defined as cessation of bleeding within ≤1 hour or between >1 and ≤4 hours after the end of infusion, respectively, with no additional coagulation products required. Hemostatic efficacy was rated poor/none if administration of other hemostatic products or red blood cells was required within 24 hours of the start of the study product infusion.^12^ In the surgery RCT, effective hemostasis was defined as intraoperative or intraprocedural blood loss not exceeding predicted blood loss by 30% or 50 mL and normal or mildly abnormal hemostasis as assessed by the surgeon, and no administration of nonstudy coagulation products.^13^ Other secondary endpoints included INR values at prespecified time points after infusion, rapid INR reduction (INR ≤1.3 at 0.5 hours after start of infusion), time to INR correction, and plasma VKDF levels at prespecified time points after infusion. Use of additional blood products and health care utilization, including LOS at ED, ICU, and general ward, were also evaluated in the acute major bleeding study. Full details of assessed outcomes in both studies have been previously described.^12^^,^^13^

### 2.3 Statistical Analysis

Hemostatic efficacy (excellent/good and poor/none) data were analyzed using logistic regression. Group differences were expressed as an odds ratio (OR) with CI representing the odds of excellent/good hemostatic efficacy in the 4F-PCC group relative to the odds in the plasma group. Treatment groups were compared using the chi-squared test.

INR ≤1.3 was analyzed using logistic regression. Due to the plasma group having the same outcomes, it was not possible to calculate an OR for this analysis, and thus, Fisher exact test was used to compare groups.

Time to INR restoration from start of infusion was analyzed using linear regression. Group differences were expressed as ORs with CI, indicating the ratio of values in the 4F-PCC group relative to values in the plasma group. Treatment groups were compared using the Mann-Whitney test.

Postinfusion INR values and VKDFs (factor II, factor VII, factor IX, and factor X) were analyzed using linear regression on a log scale in order to meet the assumptions of statistical technique. Outcomes for these data were found to have positively skewed distributions and were compared between groups using the Mann-Whitney test.

Adverse event outcomes were analyzed using logistic regression, including terms for study and patient groups. Treatment groups were compared using the chi-squared test.

Use of additional bleeding products was summarized from the bleeding study only. Median differences between groups were expressed as percentage blood product use in the 4F-PCC group minus percentage use in the plasma group. Treatment groups were compared using the chi-squared test.

Health care resource utilization data was assessed from patients who were admitted to each location. Median differences were expressed as time for the 4F-PCC group minus time for the plasma group. LOS outcomes had skewed distributions and were compared between groups using the Mann-Whitney test.

All regression analyses contained fixed effects for study and treatment. Additionally, for outcomes with a preinfusion measurement, the preinfusion value was included as a covariate in the analyses.

Initial analyses compared the overall effect of treatment for the 2 studies combined. Subsequently, the difference in the treatment effects between the 2 studies was assessed by including an interaction between study and treatment in the regression models. A significant interaction would suggest different treatment effects in the 2 studies. Where a significant interaction was found, the treatment differences were also quantified for each study separately. No separate results were shown when the interaction was not statistically significant. The final set of analyses examined additional outcomes measured in the major bleeding study only.

All statistical analyses were performed using Stata (version 15.1, StataCorp) statistical software.

## 3 Results

### 3.1 Study Population

A total of 171 patients (n = 83 received 4F-PCC; n = 88 received plasma) from 2 clinical trials, 113 patients with acute GI major bleeding and 58 patients requiring urgent GI surgery/invasive procedure, were included in this analysis.

The median dose of 4F-PCC was 2478 IU (IQR, 1876-3000 IU) and of plasma was 801 mL (IQR, 695-1000 mL). The median duration of 4F-PCC infusion was 17 minutes (IQR, 13-25 minutes) and of plasma was 150 minutes (IQR, 80-230 minutes). Study population characteristics are summarized in Table 1.

**Table 1:** Study population characteristics.

	Plasma (n = 88)	4F-PCC (n = 83)
Study, n (%)		
Major bleeding	58 (65.9)	55 (66.3)
Surgery	30 (34.1)	28 (33.7)
Procedure, n (%)^a^		
Surgery	26 (86.7)	21 (75.0)
Invasive procedure	4 (13.3)	7 (25.0)
Sex, n (%)		
Male	46 (52.3)	44 (53.1)
Female	42 (47.7)	39 (46.9)
Age (y), mean (SD)	69.9 (11.6)	70.7 (13.2)
Weight (kg), median (IQR)	75 (64-94)	80.0 (65-96)
Baseline INR, median (IQR)	3.6 (2.9-6.0)	3.7 (2.5-5.6)
Treatment duration (min), median (IQR)	150 (80-230)	17 (13-25)
4F-PCC dose (IU), median (IQR)	-	2478 (1876-3000)
Plasma dose (mL), median (IQR)	801 (695-1000)	-

4F-PCC, 4-factor prothrombin complex concentrate; INR, international normalized ratio.

a Data from surgery study only.

### 3.2 Efficacy

#### 3.2.1 Hemostatic efficacy

A hemostatic efficacy rating of excellent/good was achieved in 68 (81.9%) patients in the 4F-PCC group and in 66 (75.0%) patients in the plasma treatment group (Table 2). There was no statistically significant difference between the groups (OR, 1.52; 95% CI, 0.72-3.20), but there was a significant interaction (*P* < .005), suggesting that the group differences varied by study. Hemostatic efficacy did not vary between groups for major GI bleeding (OR, 0.93; 95% CI, 0.40-2.18). A significant difference was found for GI surgery, where 27 (96.4%) patients achieved excellent/good response with 4F-PCC compared with 22 (73.3%) in the plasma group (OR, 9.82; 95% CI, 1.14-84.6).

**Table 2:** Hemostatic efficacy.

Hemostatic efficacy, n (%)	Plasma (n = 88)	4F-PCC (n = 83)	Odds ratio^a^ (95% CI)	*P* value
Excellent/good	66 (75.0)	68 (81.9)	1.52 (0.72-3.20)	.27
Poor/none	22 (25.0)	15 (18.1)		
Hemostatic efficacy: GI bleeding, n (%)	(n = 58)	(n = 55)		
Excellent/good	44 (75.9)	41 (74.6)	0.93 (0.40-2.18)	.87
Poor/none	14 (24.1)	14 (25.4)		
Hemostatic efficacy: GI surgery, n (%)	(n = 30)	(n = 28)		
Excellent/good	22 (73.3)	27 (96.4)	9.82 (1.14-84.6)	**.04**
Poor/none	8 (26.7)	1 (3.6)		

4F-PCC, 4-factor prothrombin complex concentrate; GI, gastrointestinal.

Bold *P* value indicates a statistically significant result.

a Odds ratio indicates odds of excellent/good hemostatic efficacy in the 4F-PCC group relative to odds in the plasma group.

#### 3.2.2 Postinfusion INR values

**Figure 1:**
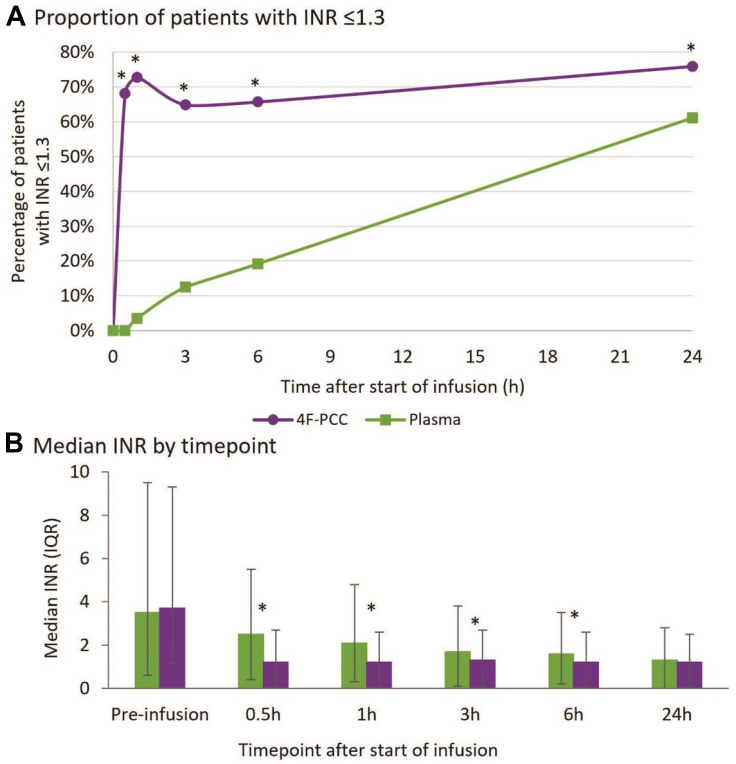
Postinfusion international normalized ratio (INR) values. (A) Proportion of patients that achieved INR correction at different time points since the start of infusion. (B) Median INR by time point; error bars indicate interquartile range. ∗*P* = .04 to <.001 as determined by linear regression analysis with fixed effects for study and treatment. Data points in A are significantly different with *P* < .001 for all time points, except for 24 hours with *P* = .04. 4F-PCC, 4-factor prothrombin complex concentrate.

Baseline median INR values were 3.7 (IQR, 2.5-5.6) and 3.5 (IQR, 2.9-6.0) for the 4F-PCC and plasma groups, respectively (OR, 0.97; 95% CI, 0.82-1.16). At 30 minutes after the start of infusion, 68.2% of patients treated with 4F-PCC had achieved an INR of ≤1.3 compared with 0.0% of patients treated with plasma (68% difference; 95% CI, 57-79; Fig 1A and Table S1). In addition, a day after the start of infusion, 75.9% of patients who received 4F-PCC and 61.2% of patients treated with plasma had an INR of ≤1.3 (14% difference; 95% CI, 0-29). Median INR was significantly lower in the 4F-PCC group than in the plasma group at all postinfusion time points up to 6 hours after start of infusion (Fig 1B and Table S2).

Rapid INR reduction 0.5 hours after the start of infusion was achieved in 53 of 83 (63.9%) patients who received 4F-PCC compared with 0 of 88 (0.0%) patients treated with plasma (*P* < .001; Table 3). Patients in the 4F-PCC group achieved INR restoration more rapidly than those in the plasma group (Table 3), with a median time to INR restoration from start of infusion of 45 minutes in the 4F-PCC group vs 1326 minutes in the plasma group (OR, 0.10; 95% CI, 0.07-0.14).

**Table 3:** International normalized ratio restoration.

	Plasma (n = 88)	4F-PCC (n = 83)	Odds ratio (95% CI)	*P* value
Rapid INR reduction 0.5 h after SoI,^a^ n (%)	0 (0.0)	53 (63.9)	-	**<.001**
Time to INR restoration from SoI^b^ (min), median (IQR)	1326 (524-1442)	45 (30-60)	0.10 (0.07-0.14)	**<.001**

4F-PCC, 4-factor prothrombin complex concentrate; INR, international normalized ratio; SoI, start of infusion.

Bold *P* value indicates a statistically significant result.

a Unable to calculate odds ratio due to all the same outcomes in the plasma group; analysis was performed using Fisher exact test.

b Ratio indicates a ratio of values in the 4F-PCC group relative to values in the plasma group.

#### 3.2.3 Postinfusion VKDF levels

**Figure 2:**
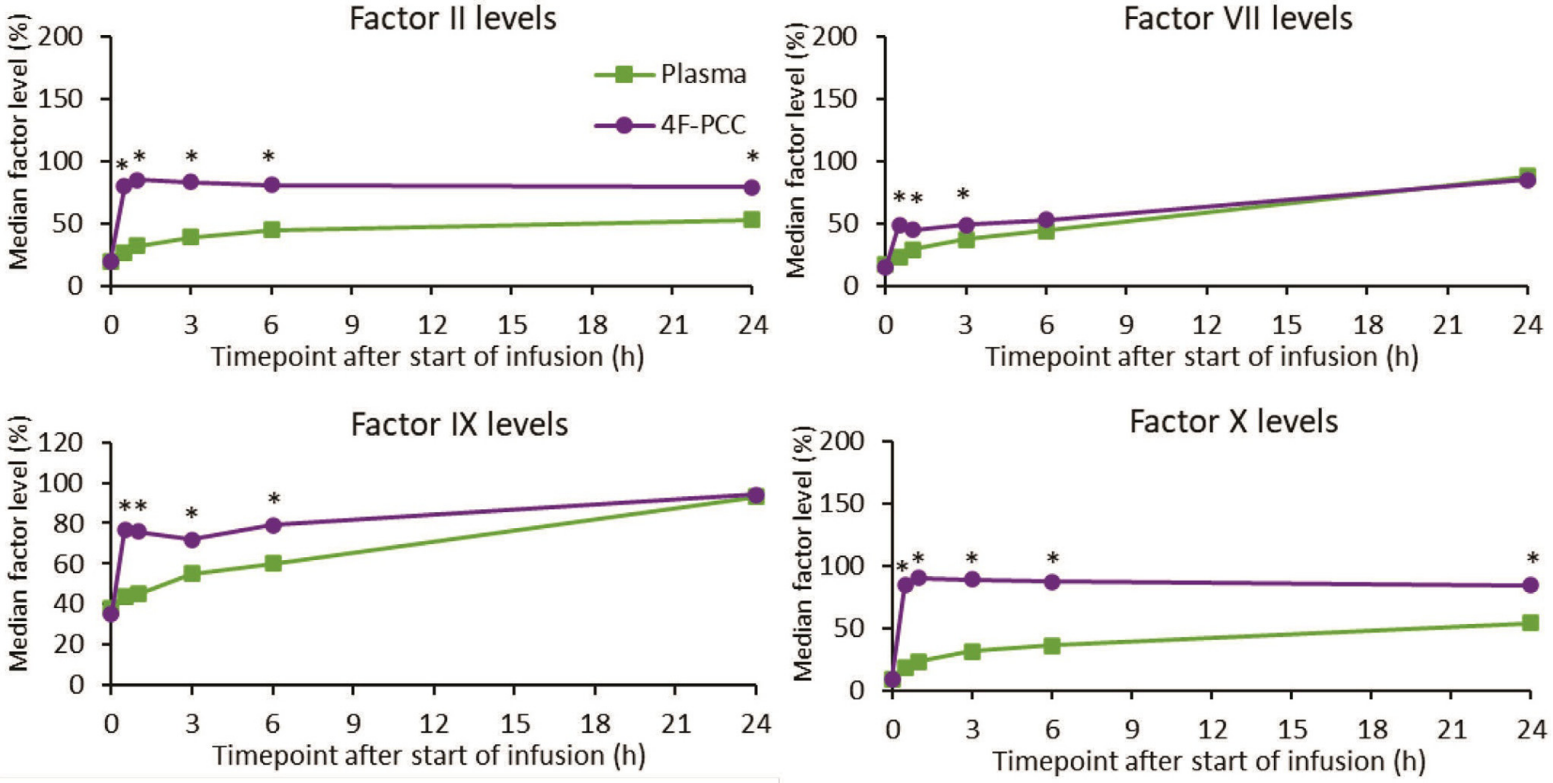
Vitamin K–dependent coagulation factor levels at different time points after the start of infusion. ∗*P* = .004 to <.001 as determined by linear regression analysis with fixed effects for study and treatment. 4F-PCC, 4-factor prothrombin complex concentrate.

All VKDF levels were significantly higher in patients treated with 4F-PCC compared with plasma up to 3 hours after the start of infusion (all *P* < .002; Fig 2 and Table S2). Median activity levels of factor II and factor X were significantly higher at all measured time points following 4F-PCC treatment compared with plasma (*P* < .001 at all time points; Fig 2 and Table S2). Thirty minutes after the start of infusion, median activity levels of all VKDFs were nearly 50% (factor VII, 49%) or exceeded 50% (factors II, IX, and X) in the 4F-PCC group (Fig 2 and Table S2). In contrast, in the plasma group, median activity levels of all VKDFs were below 50% 1 hour after the start of infusion. Median activity levels of factor IX exceeded 50% 3 hours after the start of plasma infusion, while that was not the case for factors II, VII, and X until 24 hours after the start of plasma infusion (Fig 2 and Table S2).

### 3.3 Adverse Events

No significant differences were detected in terms of incidence of thromboembolic events (OR, 1.34; 95% CI, 0.35-5.20), pulmonary edema (OR, 0.52; 95% CI, 0.09-2.91), and congestive heart failure (OR, 1.06; 95% CI, 0.25-4.42) between patients treated with 4F-PCC and those treated with plasma (Table S3). Mortality rate was also similar, with 4 of 83 (4.8%) and 6 of 88 (6.8%) deaths in the 4F-PCC and plasma groups, respectively (OR, 0.69; 95% CI, 0.19-2.55; Table S3). However, it should be noted that due to the relatively small number of adverse events, there was low statistical power to detect differences.

### 3.4 Use of Additional Blood Products

In the acute major bleeding study, no significant differences were detected in the proportion of patients requiring administration of any other blood products in the 4F-PCC and plasma groups (39 of 55 [70.9%] vs 39 of 58 [67.2%], respectively; 4% difference; 95% CI, −13 to 21; Table 4). The number of patients who required ≥1 packed red blood cell transfusion was similar in the 4F-PCC and plasma group, with no statistically significant difference (37 of 55 [67.3%] vs 37 of 58 [63.8%], respectively; 3% difference; 95% CI, −14 to 21). A total of 9 of 58 (15.5%) patients treated with plasma received additional plasma compared with 4 of 55 (7.3%) patients treated with 4F-PCC, although this difference was not statistically significant (−8% difference; 95% CI, −20 to 3).

**Table 4:** Use of additional blood products.

Blood product, n (%)	Plasma (n = 58)	4F-PCC (n = 55)	Difference % (95% CI)^a^	*P* value
Any blood product	39 (67.2)	39 (70.9)	4 (−13 to 21)	.67
pRBCs	37 (63.8)	37 (67.3)	3 (−14 to 21)	.70
RBCs	2 (3.4)	3 (5.5)	2 (−6 to 10)	.60
Platelets	0 (0.0)	1 (1.8)	2 (−2 to 5)	.30
Plasma	9 (15.5)	4 (7.3)	−8 (−20 to 3)	.17

4F-PCC, 4-factor prothrombin complex concentrate; pRBC, packed red blood cell; RBC, red blood cell.

a Group difference is expressed as the percentage blood product use in the 4F-PCC group minus the percentage used in the plasma group.

### 3.5 Health Care Resource Utilization

No patients had to be rehospitalized after treatment. There were no significant differences between patients treated with 4F-PCC and those treated with plasma in terms of median ED (1.2% difference; 95% CI, −1.0 to 3.4), ICU (−3% difference; 95% CI, −28 to 21), or general ward LOS (15% difference; 95% CI, −11 to 46) (Table S4).

## 4 Limitations

One of the limitations of this study was that it included multiple post hoc statistical analyses of data extracted from 2 prospective RCTs. In addition, since it examined only a subset of patients from 2 larger clinical trials, the relatively small sample size may have limited the ability to detect differences between groups. Another limitation was that VKDF levels represent a surrogate outcome for hemostatic efficacy; therefore, any link between VKDF levels and patient outcome should be interpreted with caution.^16^

Specifically for the acute major bleeding study, blood product transfusions were not randomized, nor were transfusion guidelines; the decision to transfuse a patient was at the discretion of the investigator and may have been influenced by personal choice or different institutional protocols.

## 5 Discussion

Overall, this study found that 4F-PCC had a rapid onset of action and was effective for VKA reversal in major GI bleeding. Compared with plasma, 4F-PCC rapidly restored INR and increased all VKDF plasma levels to at least 50% 30 minutes after the start of the infusion. Rapid INR correction and increase of VKDF plasma levels following 4F-PCC treatment may reduce the time to procedure in comparison with plasma and could subsequently improve patient outcomes. This is in line with a previous study demonstrating a significantly shorter time from start of treatment to the first procedure in VKA-treated patients with acute/severe GI bleeding following 4F-PCC treatment compared with plasma.^14^ Although a shorter time to GI procedure was not observed in the pivotal RCTs for the 4F-PCC groups, it is important to note that this might have resulted from the fact that 4F-PCCs were novel and physicians were used to plasma as the standard of care at that time, which requires several hours for INR correction.

Although plasma has been historically used for VKA reversal, studies show that restoration of INR is substantially slower with this product. Moreover, due to the low concentration of coagulation factors in plasma, large transfusion volumes are necessary to increase VKDF levels. These high volumes may increase the risk of circulatory volume overload and transfusion-related acute lung injury, the leading cause of transfusion-related morbidity and mortality, although our cohort may have been too small to demonstrate this effect.^17^, ^18^, ^19^ In contrast, 4F-PCC, a purified concentrate of VKDFs, can be administered in a smaller volume and shorter time compared with plasma, resulting in immediate restoration of VKDFs and faster correction of INR.^12^^,^^13^^,^^16^

It is important to note that although INR restoration was faster in the 4F-PCC group than in the plasma group, hemostatic efficacy was similar between groups in both RCTs. This may suggest that either a large proportion of patients with GI bleeding do well independently from the treatment received or that time to endoscopy and stopping the bleeding is more critical than time to INR restoration. The latter is in agreement with the results of a prospective single-center trial in which the only independent risk factor of all-cause mortality in high-risk patients experiencing GI bleeds was the time lapse between presentation and endoscopy.^20^ In addition, it should also be noted that some patients with massive hemorrhage may benefit from receiving plasma and/or other blood products, in addition to 4F-PCC, to provide the other coagulation factors. On the other hand, the small sample size of the current analysis may also have had an impact on the ability to detect differences between the 2 treatment groups.

In summary, 4F-PCC was associated with a nearly immediate decrease in INR and rapid VKDF restoration compared with plasma in patients with major GI bleeding or undergoing GI surgery. Yet, hemostatic efficacy was similar between the 2 groups, and therefore, larger studies might be needed to better understand patient outcomes.

## Author Contributions

MAR and JNG participated in the study concept and design and provided critical appraisal and interpretation of the data presented. Both authors provided critical appraisal and revisions of this document during its creation and reviewed and approved the final version of this manuscript prior to submission. MAR takes responsibility for the paper as a whole.

## Funding and Support

By *JACEP Open* policy, all authors are required to disclose any and all commercial, financial, and other relationships in any way related to the subject of this article as per ICMJE conflict of interest guidelines (see www.icmje.org). The authors have stated that no such relationships exist. Medical writing support was provided by Meridian HealthComms (Macclesfield, United Kingdom) in accordance with Good Publication Practice Guidelines (GPP2022) and funded by CSL Behring.

## Conflict of Interest

MAR is on the advisory committee and is a consultant for Cerus, Stago Diagnostic, CSL Behring, and CSL Plasma and has received research grants from Sysmex, Stago diagnostic, Abbott, and Hemosonic. JNG has received research support from the National Institutes of Health, Pfizer, Octapharma, and Takeda and has received consulting fees from AstraZeneca, CSL Behring, Prothya, nControl, Cayuga, and Lumosa.
