# Supplementary material for: Four-Factor Prothrombin Complex Concentrate vs Plasma in Patients on Vitamin K Antagonists With Gastrointestinal Bleeding or Needing a Gastrointestinal Procedure: A Retrospective Analysis of 2 Randomized Controlled Trials
Source: J Am Coll Emerg Physicians Open. 2025 Apr 16;6(3):100142. doi: 10.1016/j.acepjo.2025.100142 (PMC12169307; doi:10.1016/j.acepjo.2025.100142)
Supplement: Supplementary Tables 1-4 [file mmc1.docx]

**Supplementary Table 1.** Proportion of patients with INR ≤1.3 at different timepoints

| **Outcome** | **Timepoint** | **Plasma  (n=88)** | | **4F-PCC  (n=83)** | | **Difference*** **% (95% CI)** | **p-value** |
| --- | --- | --- | --- | --- | --- | --- | --- |
|  |  | **n** | **n (%)** | **n** | **n (%)** |  |  |
|  |  |  |  |  |  |  |  |
| **INR ≤1.3** | Pre-infusion | 87 | 0 (0.0) | 83 | 0 (0.0) | - | - |
|  | 30 min | 76 | 0 (0.0) | 66 | 45 (68.2) | 68 (57–79) | **<0.001** |
|  | 1 hour | 85 | 3 (3.5) | 77 | 56 (73.0) | 69 (59–80) | **<0.001** |
|  | 3 hours | 72 | 9 (12.5) | 74 | 48 (64.9) | 52 (39–65) | **<0.001** |
|  | 6 hours | 78 | 15 (19.2) | 70 | 46 (65.7) | 46 (32–60) | **<0.001** |
|  | 24 hours | 85 | 52 (61.2) | 79 | 60 (75.9) | 14 (0–29) | **0.04** |

*Difference expressed as percentage of blood product use in the 4F-PCC group minus percentage use in the plasma group.

4F-PCC, four-factor prothrombin complex concentrate; CI, confidence interval; INR, international normalized ratio.

**Supplementary Table 2.** INR and VKDF activity levels at different timepoints after start of infusion

| **Outcome** | **Timepoint** | **Plasma (n=88)** | | **4F-PCC (n=83)** | | **Ratio*** | **p-value** |
| --- | --- | --- | --- | --- | --- | --- | --- |
|  |  | **n** | **Median [IQR]** | **n** | **Median [IQR]** | **(95% CI)** |  |
|  |  |  |  |  |  |  |  |
| **INR** | Pre-infusion | 87 | 3.5 [2.9–6.0] | 83 | 3.7 [2.5–5.6] |  |  |
|  | 30 min | 76 | 2.5 [2.1–3.0] | 66 | 1.2 [1.1–1.5] | 0.52 (0.47–0.58) | **<0.001** |
|  | 1 hour | 85 | 2.1 [1.8–2.7] | 77 | 1.2 [1.1–1.4] | 0.56 (0.51–0.61) | **<0.001** |
|  | 3 hours | 72 | 1.7 [1.6–2.1] | 74 | 1.3 [1.2–1.4] | 0.73 (0.68–0.79) | **<0.001** |
|  | 6 hours | 78 | 1.6 [1.4–1.9] | 70 | 1.2 [1.1–1.4] | 0.80 (0.75–0.85) | **<0.001** |
|  | 24 hours | 85 | 1.3 [1.1–1.5] | 79 | 1.2 [1.1–1.3] | 0.96 (0.89–1.03) | 0.29 |
|  |  |  |  |  |  |  |  |
| **Factor II** | Pre-infusion | 86 | 19 [8–29] | 82 | 19 [10–28] |  |  |
|  | 30 min | 78 | 27 [20–37] | 66 | 80 [62–102] | 2.98 (2.57–3.46) | **<0.001** |
|  | 1 hour | 84 | 32 [23–41] | 79 | 85 [73–94] | 2.71 (2.41–3.05) | **<0.001** |
|  | 3 hours | 71 | 39 [32–49] | 72 | 83 [69–92] | 2.09 (1.90–2.30) | **<0.001** |
|  | 6 hours | 76 | 45 [37–51] | 72 | 81 [68–93] | 1.81 (1.63–2.02) | **<0.001** |
|  | 24 hours | 85 | 53 [45–65] | 80 | 79 [64–88] | 1.42 (1.31–1.55) | **<0.001** |
|  |  |  |  |  |  |  |  |
| **Factor VII** | Pre-infusion | 86 | 17 [8–30] | 82 | 15 [10–30] |  |  |
|  | 30 min | 78 | 23 [14–38] | 67 | 49 [39–67] | 2.09 (1.83–2.39) | **<0.001** |
|  | 1 hour | 84 | 29 [18–50] | 79 | 45 [35–65] | 1.71 (1.48–1.97) | **<0.001** |
|  | 3 hours | 72 | 37 [26–52] | 71 | 49 [36–61] | 1.29 (1.10–1.52) | **0.002** |
|  | 6 hours | 75 | 44 [32–56] | 72 | 53 [37–73] | 1.15 (0.98–1.34) | 0.09 |
|  | 24 hours | 85 | 88 [61–99] | 80 | 85 [57–101] | 0.87 (0.72–1.06) | 0.18 |
|  |  |  |  |  |  |  |  |
|  |  |  |  |  |  |  |  |
| **Factor IX** | Pre-infusion | 86 | 38 [19–57] | 82 | 35 [22–54] |  |  |
|  | 30 min | 78 | 44 [28–62] | 66 | 77 [59–96] | 1.73 (1.52–1.97) | **<0.001** |
|  | 1 hour | 84 | 45 [31–67] | 79 | 76 [55–91] | 1.66 (1.50–1.86) | **<0.001** |
|  | 3 hours | 71 | 55 [39–73] | 72 | 72 [60–89] | 1.34 (1.21–1.48) | **<0.001** |
|  | 6 hours | 76 | 60 [46–79] | 72 | 79 [68–87] | 1.20 (1.06–1.36) | **0.004** |
|  | 24 hours | 85 | 93 [73–119] | 80 | 94 [77–109] | 0.93 (0.83–1.04) | 0.22 |
|  |  |  |  |  |  |  |  |
| **Factor X** | Pre-infusion | 86 | 9 [8–16] | 82 | 9 [8–15] |  |  |
|  | 30 min | 78 | 18 [12–25] | 66 | 85 [71–112] | 4.73 (4.03–5.53) | **<0.001** |
|  | 1 hour | 84 | 23 [17–32] | 80 | 90 [76–109] | 3.93 (3.43–4.49) | **<0.001** |
|  | 3 hours | 71 | 31 [23–40] | 71 | 89 [72–106] | 2.88 (2.57–3.21) | **<0.001** |
|  | 6 hours | 76 | 36 [28–46] | 72 | 87 [71–101] | 2.38 (2.09–2.70) | **<0.001** |
|  | 24 hours | 85 | 54 [42–72] | 80 | 84 [67–96] | 1.53 (1.36–1.71) | **<0.001** |

*Ratio indicates ratio of values in the 4F-PCC group relative to values in the plasma group. Difference adjusted for pre-infusion value (where relevant).

4F-PCC, four-factor prothrombin complex concentrate; CI, confidence interval; INR, international normalized ratio; IQR, interquartile range; VKDF, vitamin-K dependent coagulation factor.

**Supplementary Table 3.** Incidence of thromboembolic events, pulmonary edema, and congestive heart failure

| **Adverse events,** n (%) | **Plasma (n=88)** | **4F-PCC (n=83)** | **Odds ratio*** **(95% CI)** | **p-value** |
| --- | --- | --- | --- | --- |
| Thromboembolic events | 4 (4.6) | 5 (6.0) | 1.34 (0.35–5.20) | 0.67 |
| Pulmonary edema | 4 (4.6) | 2 (2.4) | 0.52 (0.09–2.91) | 0.46 |
| Congestive heart failure | 4 (4.6) | 4 (4.8) | 1.06 (0.25–4.42) | 0.94 |
| Death | 6 (6.8) | 4 (4.8) | 0.69 (0.19–2.55) | 0.58 |

*Odds ratio indicates odds of adverse event in the 4F-PCC group relative to odds of adverse event in the plasma group.

4F-PCC, four-factor prothrombin complex concentrate; CI, confidence interval.

**Supplementary Table 4.** Health care resource utilization

| **Length of stay, hours** | **Plasma** | | **4F-PCC** | | **Difference* % (95% CI)** | | **p-value** | |  |  |
| --- | --- | --- | --- | --- | --- | --- | --- | --- | --- | --- |
|  | **n** | **Median (IQR)** | **n** | **Median (IQR)** |  | |  | | | |
| Emergency department | 40 | 6.0 (4.3–10.6) | 40 | 7.6 (4.7–12.2) | | 1.2 (-1.0–3.4) | | 0.24 | |  |
| Intensive care unit | 21 | 63 (42–93) | 20 | 53 (34–85) | | -3 (-28–21) | | 0.69 | |  |
| General ward | 44 | 83 (45–138) | 42 | 83 (47–152) | | 15 (-11–46) | | 0.27 | |  |

*Median difference expressed as time for the 4F-PCC group minus time for the plasma group.

4F-PCC, four-factor prothrombin complex concentrate; CI, confidence interval; IQR, interquartile range.
